# Fourier transform infrared spectroscopy as a method to study lipid accumulation in oleaginous yeasts

**DOI:** 10.1186/1754-6834-7-12

**Published:** 2014-01-23

**Authors:** Diletta Ami, Riccardo Posteri, Paolo Mereghetti, Danilo Porro, Silvia Maria Doglia, Paola Branduardi

**Affiliations:** 1 Department of Biotechnology and Biosciences, University of Milano-Bicocca, Piazza della Scienza 2, Milano 20126, Italy; 2 Department of Physics, University of Milano-Bicocca, Piazza della Scienza 3, Milano 20126, Italy; 3 Consorzio Nazionale Interuniversitario per le Scienze fisiche della Materia (CNISM) UdR Milano-Bicocca, Milano 20126, Italy; 4 Center for Nanotechnology Innovation @NEST, Italian Institute of Technology, Piazza San Silvestro 12, Pisa 56127, Italy

**Keywords:** Fourier transform infrared spectroscopy (FTIR), Principal component analysis (PCA), *Cryptococcus curvatus*, *Rhodosporidium toruloides*, *Saccharomyces cerevisiae*, Oleaginous yeasts, Fatty acids (FA), Biodiesel

## Abstract

**Background:**

Oleaginous microorganisms, such as different yeast and algal species, can represent a sustainable alternative to plant oil for the production of biodiesel. They can accumulate fatty acids (FA) up to 70% of their dry weight with a predominance of (mono)unsaturated species, similarly to what plants do, but differently from animals. In addition, their growth is not in competition either with food, feed crops, or with agricultural land.

Despite these advantages, the exploitation of the single cell oil system is still at an early developmental stage. Cultivation mode and conditions, as well as lipid extraction technologies, represent the main limitations. The monitoring of lipid accumulation in oleaginous microorganisms is consequently crucial to develop and validate new approaches, but at present the majority of the available techniques is time consuming, invasive and, when relying on lipid extraction, can be affected by FA degradation.

**Results:**

In this work the fatty acid accumulation of the oleaginous yeasts *Cryptococcus curvatus* and *Rhodosporidium toruloides* and of the non-oleaginous yeast *Saccharomyces cerevisiae* (as a negative control) was monitored *in situ* by Fourier Transform Infrared Spectroscopy (FTIR). Indeed, this spectroscopic tool can provide complementary information to those obtained by classical techniques, such as microscopy, flow cytometry and gas chromatography. As shown in this work, through the analysis of the absorption spectra of intact oleaginous microorganisms it is possible not only to monitor the progression of FA accumulation but also to identify the most represented classes of the produced lipids.

**Conclusions:**

Here we propose FTIR microspectroscopy - supported by multivariate analysis - as a fast, reliable and non invasive method to monitor and analyze FA accumulation in intact oleaginous yeasts. The results obtained by the FTIR approach were in agreement with those obtained by the other classical methods like flow cytometry and gas chromatography. Moreover, the possibility to track lipid production in real time is highly desirable to support the initial screening of strains and media as well as to optimize the scaling up experiments, which are essential for a viable and successful development of an industrial production process.

## Background

During the last two decades there has been increasing interest in biodiesel as an alternative biofuel, relying on a considerable number of research projects. In the perspective of a viable production, the use of edible vegetable oils (such as soybean and rapeseed) as well as of non-edible oils (such as *Jatropha curcas*) needs to be improved using new technologies and alternative oil sources, which are currently being explored and developed. An emerging potential alternative for biodiesel production is represented by microbial lipids (also referred to as single-cell oils (SCOs), [1]) which oleaginous microorganisms can accumulate up to 70% or more of their biomass when growing in media with a high carbon/nitrogen (C/N) ratio [2].

The applications of oleaginous fungi for biodiesel production are still few, even if they offer several advantages over conventional plant and algal resources. In particular, in comparison to open-pond grown algae and to plants, yeasts can be easily grown in bioreactors, display rapid growth rates, are unaffected by space limitations, light or climatic variations, and are also easier to scale up [3]. Moreover, oleaginous yeasts have the ability to utilize a wide range of inexpensive renewable carbon sources and while the first investigations commonly employed glucose as a carbon source, nowadays raw materials, by-products and surplus are increasingly used, leading to cost reduction and waste cutback. In particular, xylose [4,5], lactose [6], mannose, mannitol [7], and ethanol [8] have been reported as possible substrates. More recently, carbon sources obtained from lignocellulosic material have been also successfully used [9,10]. This metabolic versatility combines well with the demand for cheap production of biofuels, since the feeding substrate (and its availability) represents a relevant fraction of the overall costs. In view of developing a biorefinery-based bioprocess, nowadays most of the investigations for lipid production are focused on the selection and the development of oleaginous yeasts able to utilise glycerol - which is the major side-product of the trans-esterification of oils into biodiesel - as a carbon source for fatty acid accumulation (as examples see [11,12]).

Together with the selection of the best cell factory and of the best and cheapest medium, the development of a robust and effective production process is a primary requirement. It is consequently essential to develop a reliable and quick method for monitoring fatty acid accumulation in yeasts. It will be necessary to *i)* support the initial screening phases for strains and media, which are affected by many variables like substrate composition, carbon source, C/N ratio and temperature; *ii)* track the effective production and productivity during the first tests as well as during the initial scaling up of the process; and *iii)* further screen for improved cell factories after possible iterative trials of engineering and/or mutagenesis processes.

Different techniques for lipid quantification are now available. In particular, fluorescent microscopy, spectrophotometry or flow cytometry after staining the producing cells with specific fluorescent probes (that is, Nile red, [13]) can be used essentially for qualitative analysis. These approaches are relatively quick and do not require lipid extraction. Furthermore, thin-layer chromatography (TLC) and - more importantly - gas chromatography (GC) are qualitative and quantitative methods that can also provide information about the composition of the lipids produced by the cells, a highly desirable issue [14,15]. However, these methods are time-consuming and invasive, as they require the lipid extraction from the yeast cells, which can cause lipid losses, also due to lipase activity. Moreover, these approaches do not allow quick screening of numerous samples, or the real-time monitoring of the production process.

On the contrary, Fourier transform infrared (FTIR) spectroscopy is a non-invasive and label-free technique that allows rapid acquisition of a biochemical fingerprint of the sample under investigation, giving information on its main biomolecule content. Indeed, this spectroscopic tool is successfully applied not only to the characterization of the structural properties of isolated biomolecules, such as proteins, lipids, nucleic acids, and carbohydrates [16-21], but also to the *in situ* investigation of complex biological systems, including intact cells, tissues and whole-model organisms [22-25]. Interestingly, the FTIR method was recently used to determine the lipid accumulation in microalgae and in marine yeasts and protists [26,27]. Moreover, the use of an infrared microscope enables measurement of infrared (IR) absorption spectra from a micro-volume within the sample. In particular, adjusting the variable aperture of the microscope it is possible to select an area of interest in the sample from 250 μm × 250 μm down to approximately 20 μm × 20 μm.

We should add that the FTIR spectra of biological systems are very complex, being due to the overlapping absorption of the main biomolecules. For this reason, it is necessary to apply an appropriate multivariate analysis, able to process very high-dimensional data, to pull out the significant and non-redundant information contained in the spectra [28-30].

As previously mentioned, yeasts can be an alternative source of SCOs, and a number of different species are now under investigation. Among them, the basidiomycetes *Cryptococcus curvatus* and *Rhodosporidium toruloides* were chosen as two of the most promising cell factories [31-34]. Here we propose FTIR microspectroscopy supported by multivariate analysis as an alternative method for lipid detection *in situ* that provides not only a snapshot of FA production, but also information about the relative abundance of the most represented species. Therefore, FTIR can be proposed as a powerful tool for the initial screening and optimization and in the perspective of the bioreactor scale-up, which are all required steps for the development of a viable biodiesel production based on oleaginous microorganisms.

## Results and Discussion

### Monitoring lipid production in oleaginous yeasts by Nile red staining

**Figure 1:**
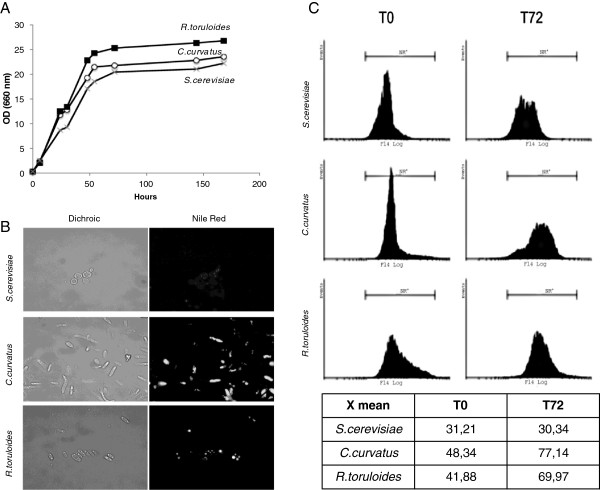
**Growth, microscopy and flow cytometry. (A)** Growth curves of *S. cerevisiae* (square), *C. curvatus* (triangle), *R. toruloides* (circle) in rich malt extract, soytone (MS) medium (30 g/l malt extract, 3 g/l soytone). **(B) ***S. cerevisiae C. curvatus* and *R. toruloides* cells were stained with Nile red (9-diethylamino-5H-benzo[α]phenoxazine-5-one) after 72 h (T72) of growth and observed under a microscope. **(C)** Flow cytometric analysis of *S. cerevisiae*, *C. curvatus* and *R. toruloides* after 0 (T0) and 72 h after inoculum in rich medium. Cells were washed twice and stained for 5 minutes with Nile red before flow cytometric analysis. X mean, values representing the medium fluorescence of *S. cerevisiae C. curvatus,* and *R. toruloides*. OD, optical density.

The growth of two oleaginous strains, *C. curvatus* and *R. toruloides*, together with the growth of the well-known non-oleaginous yeast *S. cerevisiae* was monitored measuring the cell suspension optical density over time, while the lipid accumulation was evaluated by fluorescence microscopy and flow cytometry after specific lipid staining with Nile red (Figure 1).

The strains were grown for 72 h in batch cultivation with shaking flasks in a rich medium containing malt extract and soytone (MS) as nutrients. This medium was chosen since it is known to favour lipid production in different oleaginous yeasts (see Methods for details). Samples were collected from the culture over time to monitor cellular growth, which was comparable for all the examined strains (see Figure 1A). At 0 and 72 h of growth, samples were stained with Nile red and the lipid content of the culture was evaluated by measuring the sample fluorescence by fluorescence microscopy and flow cytometry. Indeed, Nile red (9-diethylamino-5H-benzo(a)phenoxazine-5-one) is a red phenoxazine dye, present as a minor component of commercial preparations of the non-fluorescent stain Nile blue, which selectively stains lipophilic substances. Over the years, this dye has been extensively used as a lipid probe for the *in vivo* detection of intracellular lipids in intact cells by fluorescence microscopy and flow cytometry [13,35].

In this work, through dichroic optical microscopy images (Figure 1B, left panels) a profound difference was observed in the cytoplasm of the three examined yeasts. In *C. curvatus* and *R. toruloides* refractive droplets were visible, and their lipid nature was confirmed by fluorescence images (Figure 1B, right panels). On the contrary, a very faint fluorescence signal was observed in *S. cerevisiae* cells, as expected. In particular, these images enable detection of the progression and the morphology of lipid accumulation: small droplets were seen to accumulate and to collapse into bigger structures. At longer growth times most of the cells were found to be completely filled with one to two very big lipid droplets (data not shown).

The same staining procedure was applied for flow cytometric analyses. The Nile red fluorescence signals collected at 72 h from the inoculum of *C. curvatus* and *R. toruloides* were found to be higher compared to the fluorescence signals collected at time 0 h. On the contrary, this increase was found to be completely negligible in the non-oleaginous yeast *S. cerevisiae* (Figure 1C, upper panels). The mean fluorescence values for each strain at time 0 and at 72 h are reported in the table of Figure 1C. These parameters were providing a useful semiquantitative evaluation of the lipid accumulation over time for every examined yeast strain.

### Fatty acid quantification by gas chromatography

**Figure 2:**
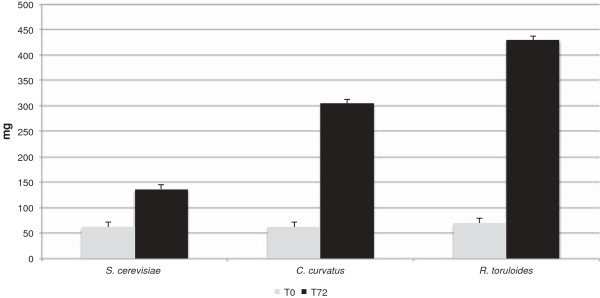
**Gas chromatography analysis. ***S. cerevisiae, C. curvatus* and *R. toruloides* total fatty acid content (mg/g biomass dry weight) extracted from 5 × 10^7^ cells at time 0 h (T0, light grey columns) and at time 72 h (T72, black columns) from the inoculum in malt extract/soytone medium.

GC is the most used technique to evaluate lipid accumulation, after the extraction of lipids from cells [32,36]. In Figure 2 the GC data of the total FA measured at time 0 (light grey columns) and 72 h (black columns) for the three strains are reported. Whereas at time 0 the total amount was similar for all the strains, after 72 h *C. curvatus* and *R. toruloides* accumulated lipid levels that were two to three times as high compared to *S. cerevisiae*. Therefore, these tests confirmed the ability of the oleaginous strains to accumulate significant amounts of lipids under the settled conditions.

### FTIR microspectroscopy to monitor lipid production in intact oleaginous yeast cells

**Figure 3:**
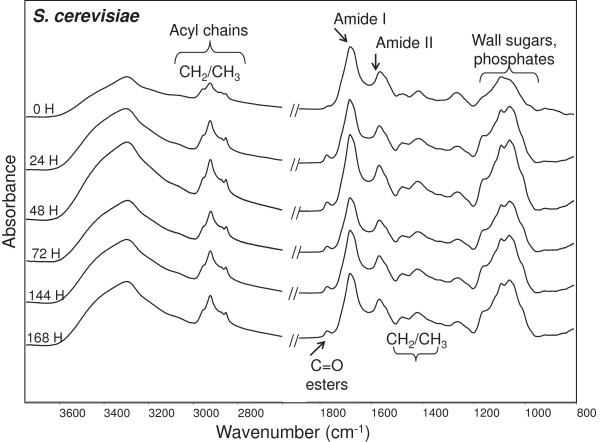
**Fourier transform infrared (FTIR) analysis of *S. cerevisiae.*** The FTIR absorption spectra of *S. cerevisiae* intact cells are reported from 0 to 168 h of growth. For comparison, spectra have been normalized to amide I band area. The most important biomolecule absorptions are indicated.

In Figure 3 the FTIR absorption spectra of intact cells of *S. cerevisiae* are reported from 0 to 168 h of growth. As expected, the spectra appear very complex, due to the absorption of the different biomolecules. In particular, the lipid acyl chains absorb between 3,030 and 2,800 cm^-1^, and 1,500 and 1,350 cm^-1^, while around 1,740 cm^-1^ the ester carbonyl IR response is observed. Moreover, between 1,700 and 1,500 cm^-1^ the spectrum is dominated by the amide I and amide II bands, respectively due to the C = O stretching and the NH bending of the peptide bond. In particular, the amide I band gives information on the protein secondary structure. It has to be mentioned that the IR absorption in the spectral range between 1,250 and 1,000 cm^-1^ is due to the contribution of nucleic acid phosphodiester groups and phospholipids, as well as to the C-O absorption of carbohydrates. Finally, between 1,000 and 800 cm^-1^ the IR response of nucleic acid and lipid moieties occurs.

When examining the spectrum time dependence of *S. cerevisiae* from 0 to 168 h (Figure 3), a few changes of the main absorption bands were observed. In particular, the intensity of the CH_2_/CH_3_ bands between 3,000 and 2,800 cm^-1^ and of the C = O absorption around 1,740 cm^-1^ was found to slightly increase, while the complex absorption between 1,250 and 1,000 cm^-1^ significantly changed. Overall, these results might reflect expected variations due to cell metabolism.

**Figure 4:**
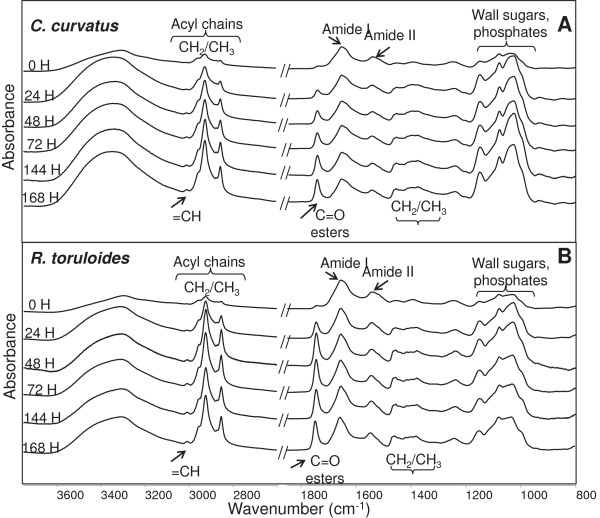
**Fourier transform infrared (FTIR) analyses of oleaginous yeasts.** The FTIR absorption spectra of *C. curvatus***(A)** and *R. toruloides* intact cells **(B)**, from 0 to 168 h of growth are reported. For comparison spectra have been normalized to the amide I band area.

Interestingly, in the case of *C. curvatus* (Figure 4A) and *R. toruloides* (Figure 4B) dramatic changes were found, mainly due to the absorption of lipids. Indeed, the intensity of the CH stretching bands between 3,050 and 2,800 cm^-1^ was found to increase from 0 up to 168 h of growth. Of particular interest is the appearance in the IR spectrum acquired at 24 h, of a component around 3,010 cm^-1^ that is due to the olefinic group =CH: this indicates the presence of unsaturated fatty acids, which is instead negligible in *S. cerevisiae* control spectrum (see Figure 3). Furthermore, in the case of *C. curvatus* (Figure 4A) and *R. toruloides* (Figure 4B) the intensity of the ester carbonyl band around 1,740 cm^-1^ was also found to increase significantly at 24 h, whereas for *S. cerevisiae* no important variations were monitored. In addition, significant changes in the absorption region between 1,250 and 1,000 cm^-1^ were observed. Remarkably, several absorption bands due to beta-glucans, which are major constituents of the yeast cell-wall, fall in this spectral region [37]. Therefore, this can reflect the important cell wall modifications that are known to occur during the production of fatty acids in the oleaginous yeasts. We should note, indeed, that the spectral changes occurring in this absorption region were more pronounced in the oleaginous yeast compared to *S. cerevisiae*.

**Figure 5:**
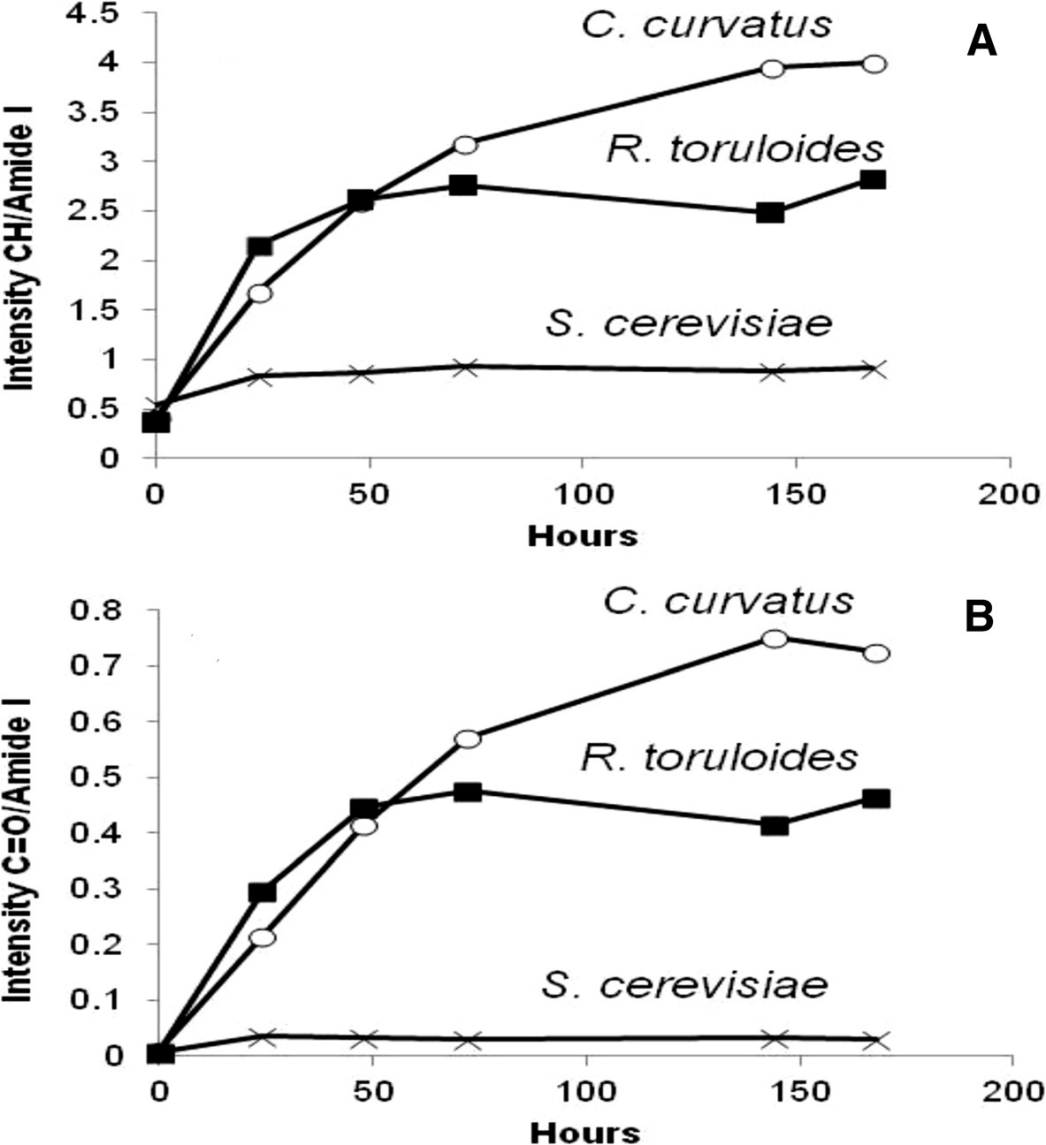
**Time dependence of fatty acid production by Fourier transform infrared (FTIR) analysis.** Time dependence of the CH stretching band area, between 3,050 and 2,800 cm^-1^, **(A)** and of the ester C = O **(B)**, normalized for the total protein content.

To better evaluate the lipid accumulation in the two producer strains, Figure 5A shows the temporal evolution of the CH stretching band area, between 3,050 and 2,800 cm^-1^, after normalization for the total protein content given by the amide I band area. As shown, *C. curvatus* and *R. toruloides* immediately start to accumulate fatty acids. In particular, *C. curvatus* reaches a higher level of lipid content compared to *R. toruloides*, as also confirmed by the temporal evolution of the ester carbonyl band area, again normalized for the total protein content (see Figure 5B).

### Second derivative analysis of the FTIR yeast spectra in the lipid absorption regions

To better evaluate the spectral changes occurring during the growth of the different studied strains, we analyzed the second derivatives of the FTIR absorption spectra, as they enable to resolve the overlapping components of the IR absorption bands [38]. In particular, we analyzed the second derivative spectra of the cells at time 0 h and we compared them with those taken at 72 h, when the oleaginous yeasts have stored an appreciable amount of FA.

**Figure 6:**
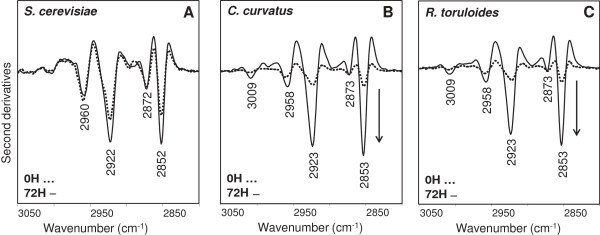
**Second derivative analyses of *S. cerevisiae*, *C. curvatus *and *R. toruloides *intact cells.** The second derivative spectra of *S. cerevisiae***(A)**, *C. curvatus***(B)** and *R. toruloides***(C)** are reported between 3,050 and 2,800 cm^-1^, after normalization at the tyrosine peak (approximately 1,516 cm^-1^). The arrows point to increasing intensities.

In Figure 6A, the second derivative spectra of *S. cerevisiae* cells are reported in the spectral range between 3,050 and 2,800 cm^-1^, dominated by the absorption of lipid acyl chains. As shown, the spectrum at time 0 h is characterized mainly by four bands, respectively at approximately 2,960 cm^-1^ (ν_asym_ CH_3_), 2,922 cm^-1^ (ν_antisym_ CH_2_), 2,872 cm^-1^ (ν_sym_ CH_3_), and 2,852 cm^-1^ (ν_sym_ CH_2_). These spectral features were found to slightly vary during *S. cerevisiae* growth, likely reflecting metabolic changes that accompany cell growth, while they dramatically change in the oleaginous yeasts *C. curvatus* and *R. toruloides* (see Figure 4A and B and Figure 6B and C). In particular, as reported in Figure 6B and C, the intensity of the acyl chain bands was found to increase from 0 to 72 h. Moreover, at 72 h a well-resolved component - almost negligible at time 0 h - appeared around 3,009 cm^-1^, due to the =CH stretching mode, typical of unsaturated fatty acids. These results, overall, indicated that the lipid content of *C. curvatus* and *R. toruloides* cells significantly changed during their growth, likely reflecting the accumulation of fatty acids at high levels, including unsaturated ones.

**Figure 7:**
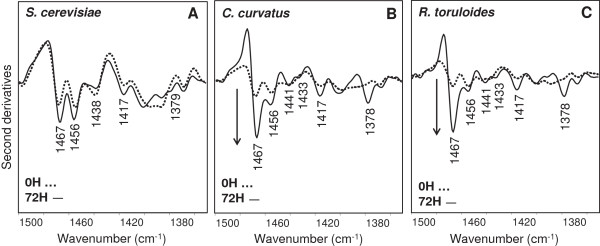
**Second derivative analyses of *S. cerevisiae*, *C. curvatus *and *R. toruloides *intact cells.** The second derivative spectra of *S. cerevisiae***(A)**, *C. curvatus***(B)** and *R. toruloides***(C)** are reported between 1,500 and 1,350 cm^-1^, after normalization at the tyrosine peak (approximately 1,516 cm^-1^). The arrows point to increasing intensities.

We then analyzed the absorption between 1,500 and 1,350 cm^-1^ (see Figure 7), where other vibrational modes due to lipid CH_2_ and CH_3_ occur. In particular, the second derivative spectrum of *S. cerevisiae* at time 0 h (Figure 7A) is characterized by four well-resolved components, around 1,467 cm^-1^ (acyl chain CH_2_ bending and/or CH_3_ deformation), 1,456 cm^-1^ and 1,438 cm^-1^ (CH_3_ deformation) [16,18,39] and 1,417 cm^-1^ (CH_2_ deformation) [40]. These spectral features were found again to slightly change during *S. cerevisiae* cell growth, and in particular in the spectrum taken at 72 h a component appears around 1,379 cm^-1^, assigned to CH_3_ bending, absent at time 0 h. As discussed for the high-frequency range, these spectral variations could reflect modifications in the metabolism accompanying cell growth. On the other hand, the spectral behaviour of *C. curvatus* and *R. toruloides*, during their growth, was found to be different compared to *S. cerevisiae*, as reported in Figure 7B and C. In particular, at 72 h, in addition to an important increase of the intensity of the 1,467 cm^-1^ band, new well-resolved components, due to CH_3_ bending vibrations, appeared at 1,441 cm^-1^, 1,433 cm^-1^, and at 1,378 cm^-1^[16]. Overall, these results confirmed that the spectral changes observed in the 3,050 to 2,800 cm^-1^ range during cell growth are common to the oleaginous yeasts.

### Principal component analysis (PCA)

With the aim to assign the spectroscopic changes observed during the growth of *C. curvatus* and *R. toruloides* to specific lipid molecules, we compared their IR response with that of standard fatty acids, chosen among the most representative products of the oleaginous yeasts [34]. To this goal, we performed the PCA that also allowed verification of the reproducibility of the data. PCA was first performed including all samples taken at 0, 24, 48 and 72 h from the inoculum. In particular, looking at the PCA score plots (Additional files 1, 2, 3 and 4), we observed a clear distinction between the time 0 h samples and the group of samples taken after 24, 48, 72 h of growth for both *C. curvatus* and *R. toruloides*. We therefore decided to proceed with our analysis, including only the samples taken at 0 and 72 h as representative of the yeast time-dependent behaviour.

**Figure 8:**
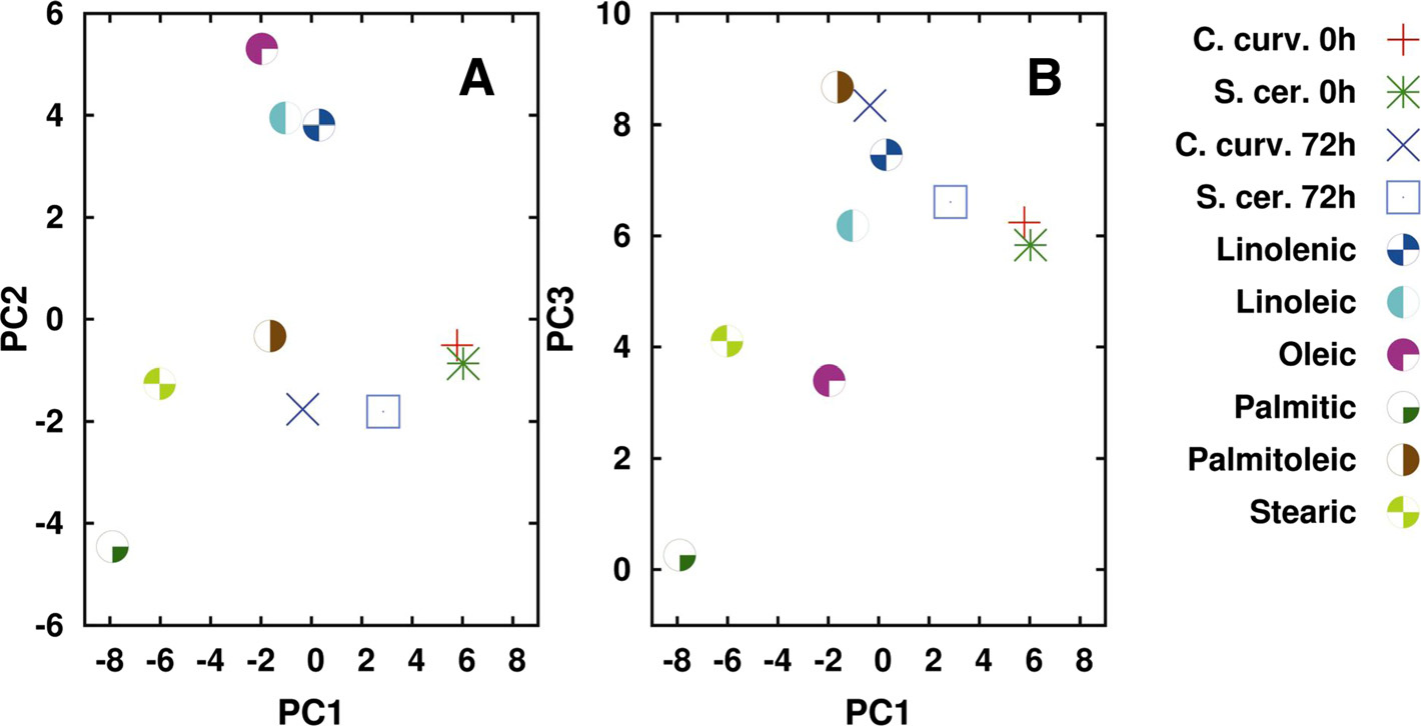
**Principal component analysis (PCA): *C. curvatus vs. S. cerevisiae.*** PCA two-dimensional score plots of *C. curvatus* and *S. cerevisiae* intact cells, and of the selected fatty-acid standards, performed between 3,050 and 2,800 cm^-1^. PC1-PC2 **(A)** and PC1-PC3 **(B)** are reported. PCA has been performed on the raw Fourier transform infrared (FTIR) spectra. For clarity, only the cluster centroid has been shown for the yeast strain data, which represents the data corresponding to three independent experiments, each one consisting of at least ten FTIR spectra.

We firstly performed the analysis between 3,050 and 2,800 cm^-1^, since this spectral range is dominated by the lipid acyl chain absorption. In Figure 8A and B we reported the PCA two-dimensional score plots obtained by analyzing the measured IR spectra of *C. curvatus* (referred to in this paragraph as the producer) and of *S. cerevisiae* (referred to in this paragraph as the control), taken at 0 and 72 h, and the selected fatty acid standards, which include saturated (palmitic and stearic acid), mono-unsaturated (oleic and palmitoleic), and poly-unsaturated (linoleic and linolenic) ones.

Interestingly, as shown in Figure 8A and B, the producer (red plus-sign) and the control (green star) strains at time 0 h almost overlap. Moreover, they are also close to the data belonging to the control strain at 72 h (blue square). On the contrary, the producer at 72 h (blue cross) is well separated from the samples at time 0 h, indicating that during growth the lipid spectral features of *C. curvatus* changed more significantly compared to the other samples.

As shown in Table 1, we then calculated the distance between each standard on one hand, and the producer and the control strains on the other, at 0 and 72 h, and their percentage variation compared to the producer and the control, respectively, taken at time 0 h. In doing this, we considered the standards displaying a distance variation of about 10% or higher as significant to determine spectral changes between the producer and the control strains.

**Table 1:** **Mean squared distances (MSD) (3,050 to 2,800 cm**^**-1**^) **for *S. cerevisiae versus C. curvatus***

**Fatty acid standard**	***S.c. *0 h**	***S.c. *72 h**	***C.c. *0 h**	***C.c. *72 h**	**Δ% *S.c.***	**Δ% *C.c.***	**Δ% (*C.c. *- *S.c.)***
Linolenic	57.31	38.8	50.2	32.25	32.3	35.75	3.45
Linoleic	73.16	48.44	66.35	37.76	33.8	43.08	9.28
Oleic	107.9	84.09	101.93	76.94	22.06	24.52	2.46
Palmitic	238	163.03	238.6	129.66	31.5	45.66	14.16
Palmitoleic	67.05	26.58	60.95	3.82	60.36	93.73	33.37
Stearic	148.49	85.46	144.61	50.47	42.45	65.1	22.65

MSD among the centroids in the three-dimensional PCA projection. Mean squared distances (MSD) among the centroids of the sets in the three-dimensional principal components analysis (PCA) projection have been reported to evaluate the distance between the producer and the control strain with the lipid standards for the range 3,050 to 2,800 cm^-1^. In addition, the percentage of the difference between the MSDs has been reported (see Methods for details). Moreover, the last column represents the difference between Δ% *C. curvatus* and Δ% *S. cerevisiae*; *S.c.*, *S. cerevisiae*; *C.c.*, *C. curvatus.*

**Figure 9:**
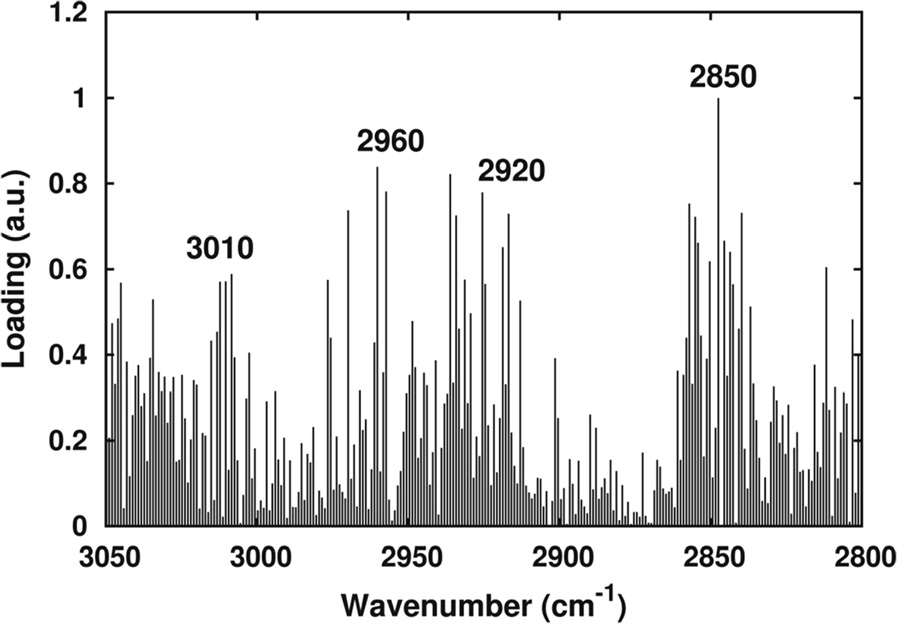
**Principal components analysis (PCA) loading plot.** Standardized PCA loadings obtained by the analysis of *C. curvatus* and *S. cerevisiae* intact cells, and of the selected fatty acid standards, between 3,050 and 2,800 cm^-1^.

First, we should note that all the analyzed standards displayed a reduction in their distances from both *S. cerevisiae* and *C. curvatus* at 72 h, compared to the two strains at time 0 h, indicating that their content increased during growth. Interestingly, we should add that this variation was found to be higher in the producer strain than in the control. However, considering the differences in the percentage variation between the control and the producer, the fatty acids mostly contributing to the changes in the spectral profiles of *C. curvatus* at 72 h in particular were palmitoleic, stearic, and palmitic. In addition, the loading plot reported in Figure 9 indicated that the spectral features contributing to the changes monitored during *C. curvatus* growth are those due to the CH_2_ stretching modes, around 2,850 cm^-1^ and 2,920 cm^-1^, followed by the CH_3_ component around 2,960 cm^-1^ and the olefinic =CH group at approximately 3,010 cm^-1^, as indicated.

To better evaluate the lipid changes occurring during yeast growth, we extended the PCA to the spectral range between 1,500 and 1,350 cm^-1^ (data not shown), where several vibrational modes due to both lipid acyl chains and head group also occur.

As reported in Table 2, we should first note that compared to the analysis performed at the higher wavenumbers, the calculated distances were found to be larger. Moreover, the differences in each standard percentage variation of the control and the producer strains were lower than those between 3,050 and 2,800 cm^-1^. Indeed, these results could be explained considering the complexity of the 1,500 and 1,350 cm^-1^ range where the IR response of other biomolecules takes place in addition to the absorption of lipids. In any case, the results (see Table 2) confirmed that all the analyzed fatty acids increased in content during cell growth of both strains, control and producer, and that the increase was again higher for the producer strain. In addition, interestingly, the analysis between 1,500 and 1,350 cm^-1^ highlighted the significant contribution of oleic acid to the new lipid-profile of the producer strain at 72 h.

**Table 2:** **Mean squared distances (MSD) (1,500 to 1,350 cm**^**-1**^) **for *S. cerevisiae versus C. curvatus***

**Fatty acid standard**	***S.c. *0 h**	***S.c. *72 h**	***C.c. *0 h**	***C.c. *72 h**	**Δ% *S.c.***	**Δ% *C.c.***	**Δ% (*C.c. *- *S.c.)***
Linolenic	301.97	161.31	296.26	134.96	46.58	54.45	7.87
Linoleic	344.05	205.9	337.59	169.46	40.16	49.8	9.64
Oleic	361.28	220.46	355.96	178.92	38.98	49.74	10.76
Palmitic	336.27	296.85	342.96	268.13	11.72	21.82	10.1
Palmitoleic	351.65	225.15	344.69	187.72	35.97	45.54	9.57
Stearic	283.68	249.18	303.72	234.04	12.16	22.94	10.78

MSD among the centroids in the three-dimensional PCA projection. Mean squared distances (MSD) among the centroids of the sets in the three-dimensional principal components analysis (PCA) projection have been reported to evaluate the distance between the producer and the control strain with the lipid standards for the range 1,500 to 1,350 cm^-1^. In addition, the percentage of the difference between the MSDs has been reported (see Methods for details). Moreover, the last column represents the difference between Δ% *C. curvatus* and Δ% *S. cerevisiae*; *S.c.*, *S. cerevisiae*; *C.c.*, *C. curvatus.*

**Figure 10:**
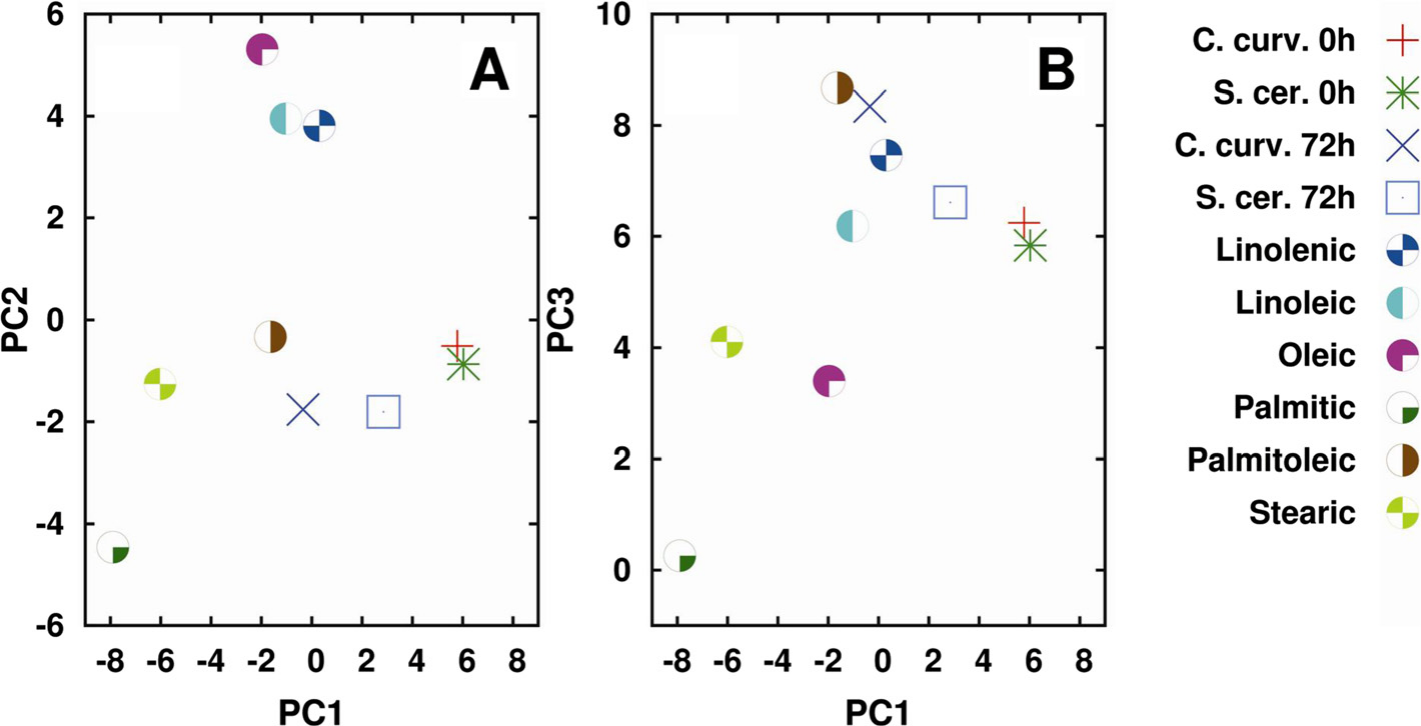
**Principal component analysis (PCA): *R. toruloides versus S. cerevisiae.*** PCA two-dimensional score plots of *R. toruloides* and *S. cerevisiae* intact cells, and of the selected fatty acid standards, performed between 3,050 and 2,800 cm^-1^. PC1-PC2 **(A)** and PC1-PC3 **(B)** are reported. PCA has been performed on the raw Fourier transform infrared (FTIR) spectra. For clarity, only the cluster centroid has been shown for the yeast strain data, which represents the data corresponding to three independent experiments, each one consisting of at least ten FTIR spectra.

**Figure 11:**
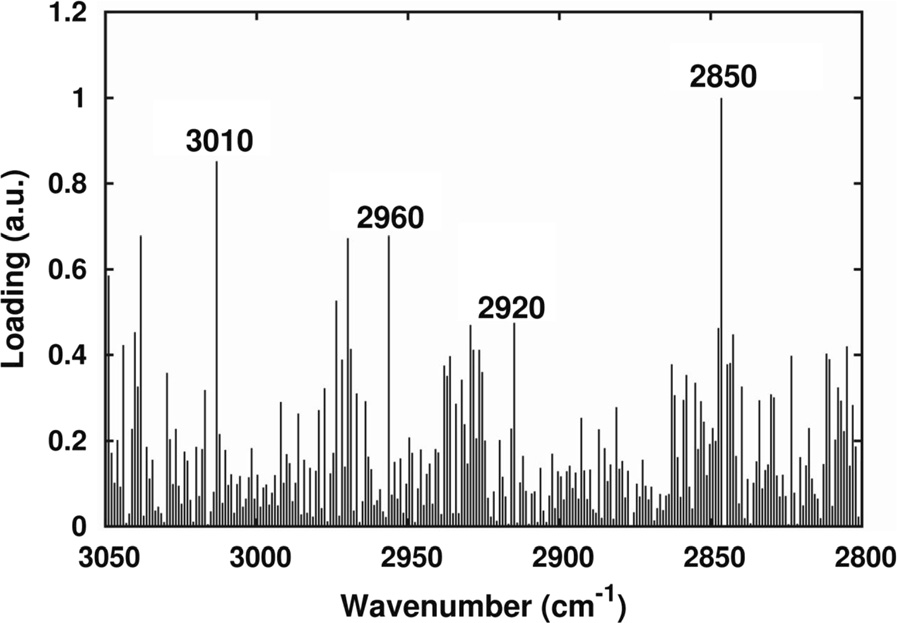
**Principal components analysis (PCA) loading plot.** Standardized PCA loadings obtained by the analysis of *R. toruloides* and *S. cerevisiae* intact cells, and of the selected fatty acid standards, between 3,050 and 2,800 cm^-1^.

As discussed for *C. curvatus*, we also performed the PCA for *R. toruloides* (Figure 10 and Table 3 referred to in this paragraph as the producer), starting from the analysis of the spectral range 3,050 to 2,800 cm^-1^. Also in this case all the analyzed standards were found to increase their content in both the control and the producer strains during cell growth, and again, the increase was higher for the oleaginous strain (Table 3). In particular, the fatty acids mostly contributing to the spectral changes observed during growth were palmitoleic, stearic, and palmitic. We should add that linoleic and oleic acids were also found to contribute (even if to a minor extent compared to the above lipid molecules) to the producer lipid profile at 72 h, as indicated by the differences in their percentage variation, respectively, of about 15 and 10%. Interestingly, as reported in the loading plot of Figure 11, the spectral components mostly contributing to the observed variance were in particular those due to the CH_2_ (ν_sym_) and the =CH, likely reflecting a higher amount of unsaturated fatty acids in *R. toruloides*, compared to *C. curvatus* (see Figure 9).

**Table 3:** **Mean squared distances (MSD) (3,050 and 2,800 cm**^**-1**^**) *S. cerevisiae versus R. toruloides***

**Fatty acid standard**	***S.c. *0 h**	***S.c. *72 h**	***R.t. *0 h**	***R.t. *72 h**	**Δ% *S.c.***	**Δ% *R.t.***	**Δ% (*R.t. *- *S.c.)***
Linolenic	55.82	37.04	61.67	36.66	33.65	40.56	6.91
Linoleic	73.01	47.99	78.78	39.54	34.27	49.81	15.54
Oleic	108.69	84.59	112.58	75.89	22.18	32.59	10.41
Palmitic	239.4	165.89	245.62	111.58	30.7	54.57	23.87
Palmitoleic	66.72	25.81	75.69	3.23	61.32	95.74	34.42
Stearic	149.02	86.1	157.31	38.09	42.22	75.78	33.56

MSD among the centroids in the three-dimensional PCA projection. Mean squared distances (MSD) among the centroids of the sets in the three-dimensional principal components analysis (PCA) projection have been reported to evaluate the distance between the producer and the control strain with the lipid standards for the range 3,050 to 2,800 cm^-1^. The percentage of the difference between the MSDs has been also reported (see Methods for details). Moreover, the last column represents the difference between Δ% *R. toruloides* and Δ% *S. cerevisiae*; *S.c.*, *S. cerevisiae*; *R.t.*, *R. toruloides.*

We then extended the PCA to the range between 1,500 and 1,350 cm^-1^ (data not shown) and interestingly we again found some results for *R. toruloides* (Table 4) that were slightly different from those observed for *C. curvatus*. In particular, for *R. toruloides* the differences in the percentage variation of all the analyzed standards were significant (*that is,* higher than 10%), thus suggesting that *R. toruloides* produced a wider variety of fatty acids compared to *C. curvatus, which* in turn meant it was more selective. Of note, we should add that the fatty acids mainly produced by *C. curvatus* belong to saturated (palmitic and stearic) and mono-unsaturated ones (palmitoleic and oleic), thus representing good candidates for the production of biofuel.

**Table 4:** **Mean squared distances (MSD) (1,500 and 1,350 cm**^**-1**^**) *S. cerevisiae versus R. toruloides***

**Fatty acid standard**	***S.c. *0 h**	***S.c. *72 h**	***R.t. *0 h**	***R.t. *72 h**	**Δ% *S.c.***	**Δ% *R.t.***	**Δ% (*R.t. *- *S.c.)***
Linolenic	292.12	151.72	299.24	84.48	48.06	71.77	23.71
Linoleic	341.28	202.45	332.15	121.66	40.68	63.37	22.69
Oleic	362.75	221.33	349.84	135.73	38.99	61.2	22.21
Palmitic	326.31	284.29	322.42	233.57	12.88	27.56	14.68
Palmitoleic	351.93	224.39	334.66	140.91	36.24	57.89	21.65
Stearic	265.9	229.43	311.99	216.98	13.71	30.45	16.74

MSD among the centroids in the three-dimensional PCA projection. Mean squared distances (MSD) among the centroids of the sets in the three-dimensional principal components analysis (PCA) projection have been reported to evaluate the distance between the producer and the control strain with the lipid standards for the range 1,500 and 1,350 cm^-1^. In addition, the percentage of the difference between the MSDs has been reported (see Methods for details). Moreover, the last column represents the difference between Δ% *R. toruloides* and Δ% *S. cerevisiae*; *S.c.*, *S. cerevisiae*; *R.t.*, *R. toruloides.*

## Conclusions

In this work we have reported the use of FTIR microspectroscopy as a powerful tool to monitor *in situ* fatty acid accumulation over time in oleaginous yeasts. In particular, we monitored the lipid production in two oleaginous yeasts taken as model systems, using the FTIR approach. Moreover, with the support of PCA we were able to obtain information about the FA profile of the oleaginous yeasts. Interestingly, our results were in excellent agreement with those reported in the literature [33,41]. In particular, we should underline that the FTIR analysis is a fast and non-invasive technique that does not require cell disruption and/or lipid extraction. This is indeed an important asset, as not only does it decrease the time and costs associated with the currently used methods (*that is,* GC), but it also reduces the risk of lipid loss and degradation. Of note, this method, supported by multivariate analysis, can provide not only a qualitative output of lipids, but it can also discriminate among the different classes of the produced FA, enabling the optimization of the production process for matching the FA profile with the requirements of the applications of interest. Finally, a further outcome of great general and applicative interest of our FTIR study has been the detection of cell-wall modifications occurring during FA accumulation. Indeed, it is well-known that one of the main drawbacks of biodiesel production from oleaginous yeasts is represented by the downstream process, lipid extraction being one of the determining steps. Product extraction from cells is generally detrimental in terms of yields and costs, but in this case it is particularly tedious, as the cell wall of oleaginous yeasts becomes more and more difficult to break while cells are accumulating FA. Considering the limited information on the genome of these yeasts and the even more limited tools for their manipulation, FTIR could be a useful support in the screening of possible cell-wall mutants obtained by indirect engineering approaches.

## Methods

### Strains and growth conditions

*C. curvatus* DSMZ 70022 and *R. toruloides* DSMZ 4444 were purchased from DSMZ [42]. Strains were stored at −80°C in 20% glycerol. The *S. cerevisiae* strain used in this study was GRF18U (*MATa; ura3; leu2-3,112; his3-11,15; cir*^*+*^, [43]). Yeasts were cultivated either in YPD (10 g/l yeast extract, 20 g/l Tryptone, 20 g/l D-glucose) or in MS (30 g/l malt extract, 3 g/l soytone) media, the second being preferred for lipid accumulation, according to the provider’s indication (http://www.dsmz.de/microorganisms/medium/pdf/DSMZ_Medium90.pdf). YPD medium was utilised only for control experiments (not shown). Cultivations were performed in 250-ml shake flasks with 50 ml of media, at 25°C and 220 rpm. Growth was monitored by measuring the optical density at 660 nm (OD660) in a Shimadzu UV-1800 UV spectrophotometer (Shimadzu Corporation).

Yeast extract and soytone were provided by Biolife Italiana S.r.l., Milano, Italia. Tryptone and malt extract were provided by Dyfco, NJ, USA. D-glucose and glycerol were provided by Aldrich Co., St Louis, MO, USA.

### Fluorescence microscopy and citofluorimetry

Cell staining for lipids analysis was performed by using Nile red (Sigma Aldrich Co., St Louis, MO, USA) at a final concentration of 31.4 μM in PBS buffer (0.05 M, pH 7.0). A Nile red stock solution (314 μM) was prepared by dissolving Nile red powder in acetone. Before measurements, cells were incubated for 5 minutes in the dark at room temperature.

Fluorescence microscopy studies were carried out with a Nikon Eclypse E600 (Nikon Instruments, Inc.). Nile red fluorescence was registered with two spectral settings: yellow-gold fluorescence, using a 450- to 500-nm band-pass exciter filter and red fluorescence using a 515- to 560-nm band-pass exciter filter. Images of stained cells were acquired both in dichroic and fluorescence mode.

Flow cytometry was conducted using a Beckman Coulter Cytomics FC500 (Beckman Coulter, Inc). A total of 20,000 cells were measured for each sample (FL4 channel 575 nm +/− 15 nm). Data analysis was performed afterwards with WinMDI 2.8 software, build #13 01-19-2000 (Purdue University, Cytometry Laboratories http://facs.scripps.edu/software.html).

### Gas chromatography analysis

To determine the lipid content in yeast cells, lipids were extracted, based on the method of Bligh and Dyer [44] with modifications and then analyzed through GC. Briefly, 10 OD (about 5 × 10^8^ cells) of samples was centrifuged at 4,000 rpm for 10 minutes; cells were then washed twice with 1 ml of distilled water. Pellets were then resuspended in 5 ml of MeOH/CHCl_3_ (2:1) and mechanically disrupted twice using French Press at 28,000 psi (Constant Cell Disruption System, Constant System Ltd). Then, 2 ml of citric acid and 3 ml of CHCl_3_ were added to the samples. After mixing, the samples were centrifuged at 4,000 rpm for 2 minutes and the upper phase was discarded. Derivation of methyl esters from fatty acids was as previously described [45]. Fatty-acid methyl esters were analyzed with a GC-mass spectrometer (GC-MS) (Agilent 7920A. Agilent J&W column, 30 m in length with an internal diameter of 0.25 mm and a film thickness of 0.25 mm, J&W Scientific, Rancho Cordova; Waters mass spectrometer 4 m). Decanoic acid was used as an internal standard.

### FTIR microspectroscopy

Yeast cells from *S. cerevisiae*, *C. curvatus*, and *R. toruloides*, at 0, 24, 48, 72, 144, and 168 h of growth were washed three times in distilled water to eliminate medium contamination. Approximately 3 μl of the cell suspensions were then deposited onto an IR transparent BaF_2_ support, and dried at room temperature for about 30 minutes to eliminate the excess water.

FTIR absorption spectra were acquired in transmission mode, between 4,000 and 700 cm^-1^, by means of a Varian 610-IR infrared microscope coupled to the Varian 670-IR FTIR spectrometer (both from Varian Australia Pty Ltd), equipped with a mercury cadmium telluride (MCT) nitrogen-cooled detector. The variable microscope aperture was adjusted from approximately 60 μm × 60 μm to 100 μm × 100 μm). Measurements were performed at 2 cm^-1^ spectral resolution; 25 KHz scan speed, triangular apodization, and by the accumulation of 512 scan co-additions. When necessary, spectra were corrected for residual water vapour absorption [46,47].

Spectral analysis was conducted in the spectral range between 4,000 and 800 cm^−1^. To this aim, second-derivative spectra were obtained following the Savitsky-Golay method (third-grade polynomial, 9 smoothing points), after a binomial 13 smoothing points of the measured spectra, using the GRAMS/32 software (Galactic Industries Corporation, USA).

To verify the reproducibility and reliability of the spectral results, more than three independent preparations were analyzed.

### Principal component analysis of FTIR data

Multivariate statistical analysis of the measured spectra were performed using Octave version 3.2.4 [48]. Data were first preprocessed using *z*-score normalization, that is, spectra were centred by their mean and divided by their standard deviation. The covariance matrix has been computed and diagonalized to obtain eigenvectors sorted according to the magnitude of the corresponding eigenvalues [49]. For both *C. curvatus* and *R. toruloides* the first three eigenvectors already describe approximately 96% and 91% of the total variance for the spectral ranges 1,500 to 1,350 cm^-1^ and 3,050 to 2,800 cm^-1^, respectively. A set of principal components has been obtained projecting the original spectra on the subspace defined by the first three eigenvectors. To quantify the distance from the lipid standards, the mean squared distance (MSD) among the centroids of the clusters in the three-dimensional PCA projection has been measured as follows:

$$\text{MSD}\left(K^{t},S\right)=\frac{1}{3}{\sum_{\text{pc}=1}^{\text{pc}=3}\left(C_{\mathrm{pc}}\left(K^{t}\right)-C_{\text{pc}}\left(S\right)\right)}^{2}$$

where *K* is the sample (*C. curvatus*, *R. toruloides*, *S. cerevisiae*), *t* corresponds to the time of growth (0 h, 72 h), S indicates the standard lipids, *pc* is the principal component index and *C*_*pc*_ is the centroid of the cluster. The centroid has been computed as the median value among the replicas within the same set of data.

Moreover, to better quantify how much samples differ in terms of distance with the standard lipids, the percentage of the difference between the MSDs has been computed as:

$$\Delta \left(K^{72h},K^{\text{Oh}}\right)\%=\left[1\frac{\text{MSD}\left({K^{72}}^{h},S\right)}{\text{MSD}\left({K^{O}}^{h},S\right)}\right]*100$$

A positive value indicates that a given lipid standard contributes to the spectral profile changes of the sample at 72 h compared to the time 0 h, indicating that it is accumulated during the yeast growth.

## Abbreviations

C/N: carbon/nitrogen; FA: fatty acid; FTIR: Fourier transform infrared; GC: gas chromatography; IR: infrared; MCT: mercury cadmium telluride; MS: malt extract, soytone; PCA: principal component analysis; MSD: mean squared distance; OD: optical density; PBS: phosphate-buffered saline; SCO: single cell oil; TLC: thin-layer chromatography; YPD: yeast extract, peptone, dextrose.

## Competing interests

The authors declare that they have no competing interests.

## Authors’ contributions

RP carried out the shake-flasks experiments for lipid production and the fluorescent staining and GC analyses, participated in the evaluation of the data and in compiling the manuscript. DA carried out the FTIR analyses, collaborated with the PCA analysis, participated in the evaluation of the data and in compiling the manuscript. PM performed the PCA analysis of the FTIR data. DP participated in the experimental work design and contributed to the data interpretation. SMD and PB conceived the study, participated in its design and compiled the manuscript. All the authors have read and approved the final manuscript.

## Supplementary Material

Additional file 1: Figure S1 Principal component analysis (PCA) of yeast cells at 0, 24, 48 and 72 h of growth: *C. curvatus* versus *S. cerevisiae*. PCA 2D-score plots of *C. curvatus* and *S. cerevisiae* intact cells, performed between 3,050 and 2,800 cm^-1^. All samples taken at 0, 24, 48 and 72 h were included in the analysis. PC1-PC2 (A) and PC1-PC3 (B) are reported. PCA has been performed on the raw Fourier transform infrared (FTIR) spectra. Click here for file

Additional file 2: Figure S2 Principal component analysis (PCA) of yeast cells at 0, 24, 48 and 72 h of growth: *R. toruloides* versus *S. cerevisiae*. PCA two-dimensional score plots of *R. toruloides* and *S. cerevisiae* intact cells, performed between 3,050 and 2,800 cm^-1^. All samples taken at 0, 24, 48 and 72 h were included in the analysis. PC1-PC2 (A) and PC1-PC3 (B) are reported. PCA has been performed on the raw Fourier transform infrared (FTIR) spectra. Click here for file

Additional file 3: Figure S3 Principal component analysis (PCA) of yeast cells at 0, 24, 48 and 72 h of growth: *C. curvatus* versus *S. cerevisiae.* PCA two-dimensional score plots of *C. curvatus* and *S. cerevisiae* intact cells, performed between 1,500 and 1,350 cm^-1^. All samples taken at 0, 24, 48 and 72 h were included in the analysis. PC1-PC2 (A) and PC1-PC3 (B) are reported. PCA has been performed on the raw Fourier transform infrared (FTIR) spectra. Click here for file

Additional file 4: Figure S4 Principal component analysis (PCA) of yeast cells at 0, 24, 48 and 72 h of growth: *R. toruloides* versus *S. cerevisiae.* PCA two-dimensional score plots of *R. toruloides* and *S. cerevisiae* intact cells, performed between 1,500 and 1,350 cm^-1^. All samples taken at 0, 24, 48 and 72 h were included in the analysis. PC1-PC2 (A) and PC1-PC3 (B) are reported. PCA has been performed on the raw Fourier transform infrared (FTIR) spectra. Click here for file
