# Supplementary material for: Fourier transform infrared spectroscopy as a method to study lipid accumulation in oleaginous yeasts
Source: Biotechnol Biofuels. 2014 Jan 23;7:12. doi: 10.1186/1754-6834-7-12 (PMC3923900; doi:10.1186/1754-6834-7-12)
Supplement: Additional file 2: Figure S2 — Principal component analysis (PCA) of yeast cells at 0, 24, 48 and 72 h of growth: R. toruloides versus S. cerevisiae. PCA two-dimensional score plots of R. toruloides and S. cerevisiae intact cells, performed between 3,050 and 2,800 cm-1. All samples taken at 0, 24, 48 and 72 h were included in the analysis. PC1-PC2 (A) and PC1-PC3 (B) are reported. PCA has been performed on the raw Fourier transform infrared (FTIR) spectra. [file 1754-6834-7-12-S2.pdf]

Figure S2

Principal component analysis of yeast cells at 0, 24, 48 and 72 hours of growth: *R. toruloides vs. S. cerevisiae*

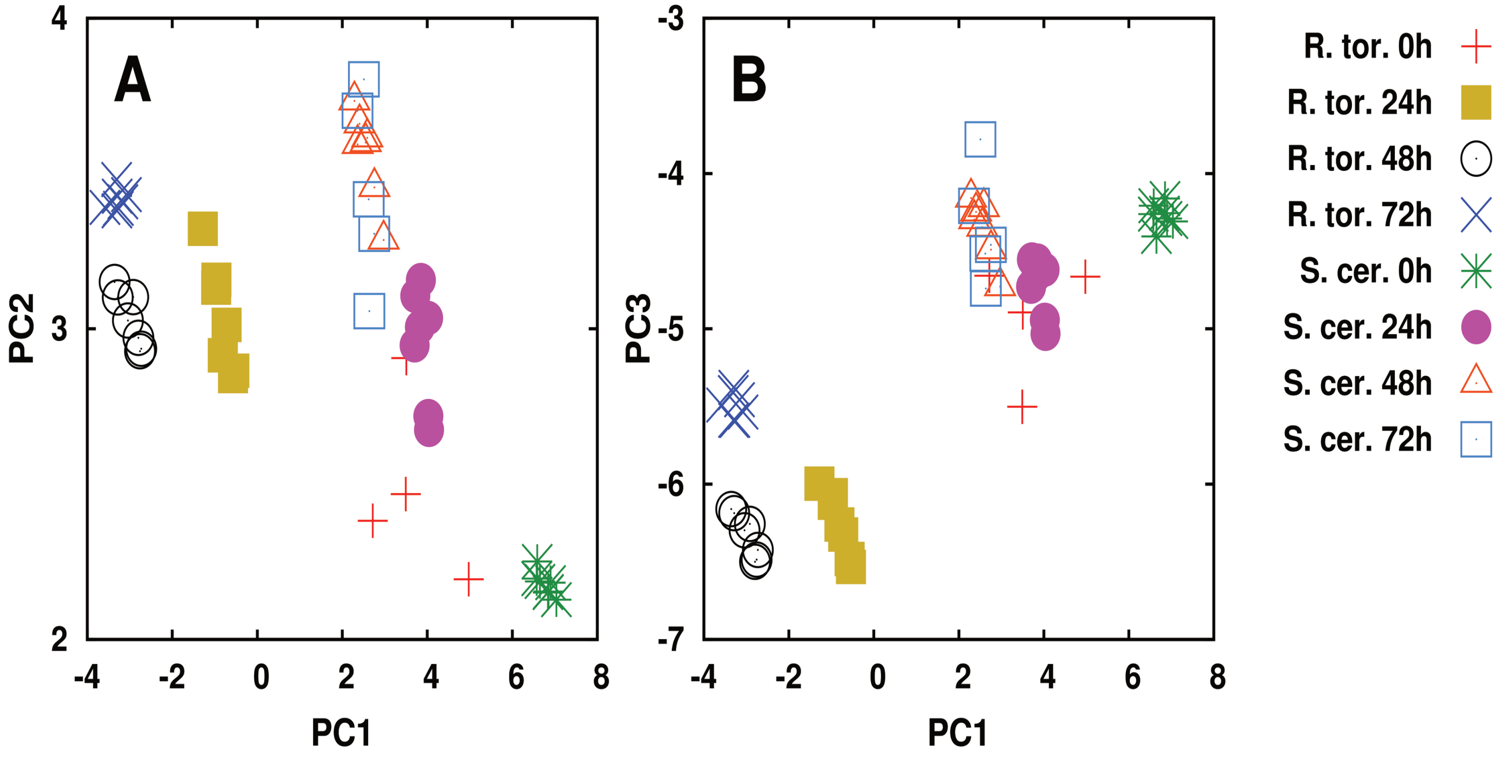

PCA 2D-score plots of *R. toruloides* and *S. cerevisiae* intact cells, performed between 3050 - 2800 cm^{-1}. All samples taken at 0, 24, 48 and 72 hours were included in the analysis. PC1-PC2 (A) and PC1-PC3 (B) are reported. PCA has been performed on the raw FTIR spectra.
