# Supplementary material for: Fourier transform infrared spectroscopy as a method to study lipid accumulation in oleaginous yeasts
Source: Biotechnol Biofuels. 2014 Jan 23;7:12. doi: 10.1186/1754-6834-7-12 (PMC3923900; doi:10.1186/1754-6834-7-12)
Supplement: Additional file 3: Figure S3 — Principal component analysis (PCA) of yeast cells at 0, 24, 48 and 72 h of growth: C. curvatus versus S. cerevisiae. PCA two-dimensional score plots of C. curvatus and S. cerevisiae intact cells, performed between 1,500 and 1,350 cm-1. All samples taken at 0, 24, 48 and 72 h were included in the analysis. PC1-PC2 (A) and PC1-PC3 (B) are reported. PCA has been performed on the raw Fourier transform infrared (FTIR) spectra. [file 1754-6834-7-12-S3.pdf]

Figure S3

Principal component analysis of yeast cells at 0, 24, 48 and 72 hours of growth:
C. curvatus vs. S. cerevisiae

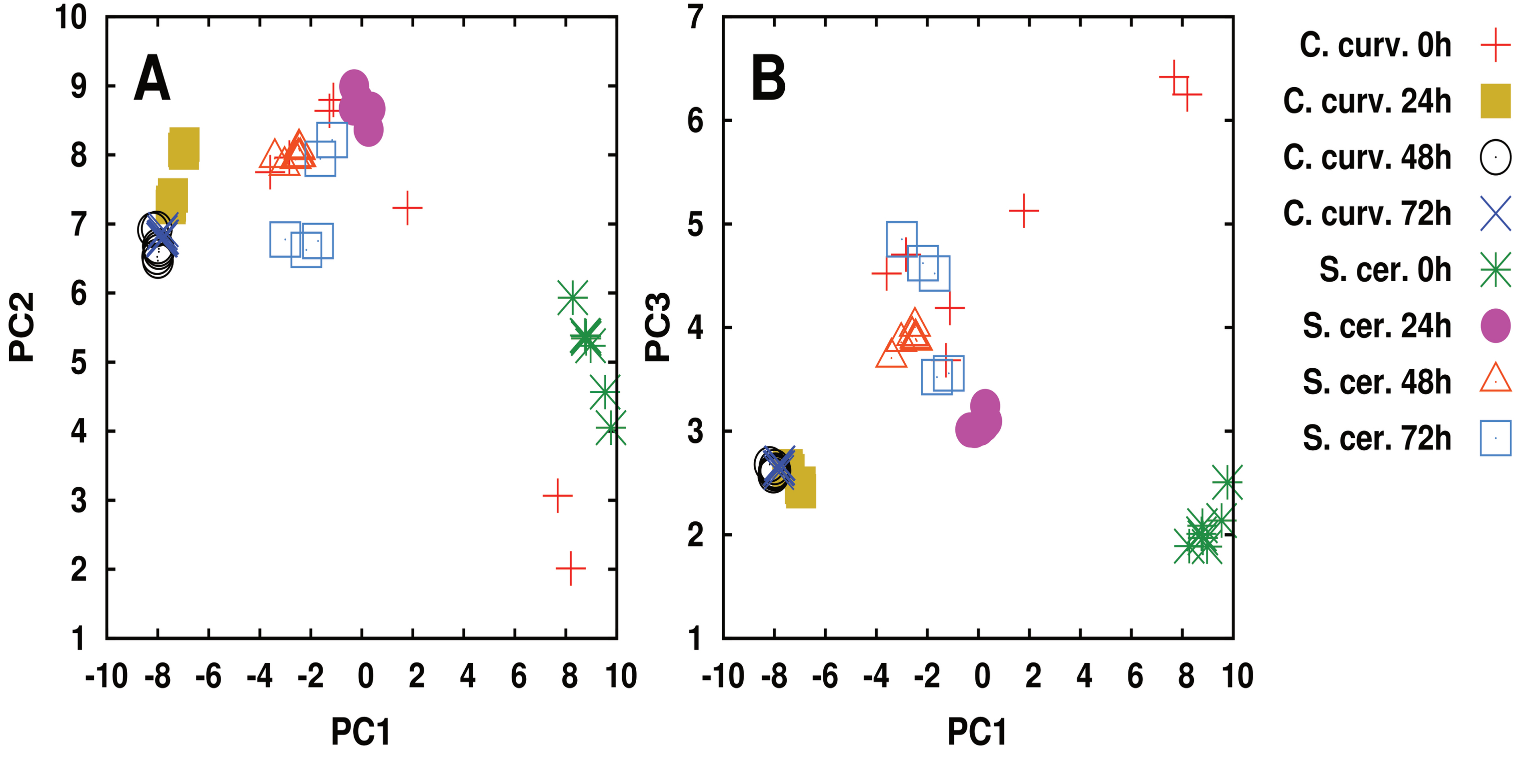

PCA 2D-score plots of *C. curvatus* and *S. cerevisiae* intact cells, performed between 1500 - 1350 cm^{-1}. All samples taken at 0, 24, 48 and 72 hours were included in the analysis. PC1-PC2 (A) and PC1-PC3 (B) are reported. PCA has been performed on the raw FTIR spectra.
